# Two waves of colonization straddling the K–Pg boundary formed the modern reef fish fauna

**DOI:** 10.1098/rspb.2014.0321

**Published:** 2014-05-22

**Authors:** S. A. Price, L. Schmitz, C. E. Oufiero, R. I. Eytan, A. Dornburg, W. L. Smith, M. Friedman, T. J. Near, P. C. Wainwright

**Affiliations:** 1 Department of Evolution and Ecology, University of California, Davis, CA 95618, USA; 2 W. M. Keck Science Department, Claremont McKenna, Pitzer, and Scripps Colleges, 925 North Mills Avenue, Claremont, CA 91711, USA; 3 Department of Biological Science, Towson University, Towson, MD 21252, USA; 4 Department of Ecology and Evolutionary Biology and Peabody Museum of Natural History, Yale University, New Haven, CT, USA; 5 Department of Ecology and Evolutionary Biology and Biodiversity Institute, University of Kansas, Lawrence, KS 66045, USA; 6 Department of Earth Sciences, University of Oxford, Oxford OX1 3AN, UK

**Keywords:** macroevolution, reef fishes, Cretaceous–Palaeogene mass extinction, niche-filling models

## Abstract

Living reef fishes are one of the most diverse vertebrate assemblages on Earth. Despite its prominence and ecological importance, the origins and assembly of the reef fish fauna is poorly described. A patchy fossil record suggests that the major colonization of reef habitats must have occurred in the Late Cretaceous and early Palaeogene, with the earliest known modern fossil coral reef fish assemblage dated to 50 Ma. Using a phylogenetic approach, we analysed the early evolutionary dynamics of modern reef fishes. We find that reef lineages successively colonized reef habitats throughout the Late Cretaceous and early Palaeogene. Two waves of invasion were accompanied by increasing morphological convergence: one in the Late Cretaceous from 90 to 72 Ma and the other immediately following the end-Cretaceous mass extinction. The surge in reef invasions after the Cretaceous–Palaeogene boundary continued for 10 Myr, after which the pace of transitions to reef habitats slowed. Combined, these patterns match a classic niche-filling scenario: early transitions to reefs were made rapidly by morphologically distinct lineages and were followed by a decrease in the rate of invasions and eventual saturation of morphospace. Major alterations in reef composition, distribution and abundance, along with shifts in climate and oceanic currents, occurred during the Late Cretaceous and early Palaeogene interval. A causal mechanism between these changes and concurrent episodes of reef invasion remains obscure, but what is clear is that the broad framework of the modern reef fish fauna was in place within 10 Myr of the end-Cretaceous extinction.

## 1. Introduction

Despite the highly fragmented distribution of reefs globally, reef fish faunas are remarkably similar; characteristic lineages are abundant on reefs around the world, such as wrasses, damselfishes, tangs and butterflyfishes on coral reefs [1] and porgies, rockfishes and wrasses on temperate rocky reefs [2,3]. These reef fishes are integral to the regulation and maintenance of the reef ecosystem through nutrient cycling [4], bio-erosion [5], herbivory and predation [6]. The evolutionary histories of fishes and modern reefs are therefore strongly interconnected but the evolutionary formation of the modern reef fish fauna remains poorly understood.

The spectacular diversity of fishes on reefs has been attributed to the supposed stability of reefs over long stretches of geological time [7], but reefs changed rapidly and dramatically in composition, abundance and geographical distribution during the early evolution of modern reef fishes [8]. Reefs are commonly defined as ‘as laterally confined structures developed by the growth or metabolic activity of sessile benthic aquatic organisms’ [9, p. 3] and modern reefs consist both of tropical coral reefs and temperate rocky reefs dominated by algae. In the Late Cretaceous (*ca* 100–66 Myr ago), reefs were primarily single-layer beds or mounds formed by corals, skeletal sponges and rudist bivalves [10]. Coral reef volume declined throughout much of the Cretaceous and rudist bivalves were dominant [11], although it has been argued that these molluscs were not true reef-builders [12]. The end-Cretaceous mass extinction (Cretaceous–Palaeogene; K–Pg) did not result in a decline of reef volume, but it did result in the loss of diversity [13,14]: rudists went extinct at, or close to, the K–Pg (66 Ma) [10] along with an estimated 45% of all scleractinian coral species [13]. Although reef ecosystems rebuilt slowly afterwards [15], coral diversity recovered fairly rapidly from the K–Pg [13] and coral formations were more geographically widespread in the earliest Palaeogene than in the Late Cretaceous [15,16]. A substantial drop in reef volume almost 10 Myr after the K–Pg [14] may have been triggered by ocean acidification [17] during the Palaeocene–Eocene thermal maximum (PETM) [14]. From the mid-Eocene (*ca* 45 Ma) onwards, coral-dominated framework reefs became the most common reef type [15] and spread globally from the late Eocene into the Oligocene [8].

**Figure 1.**
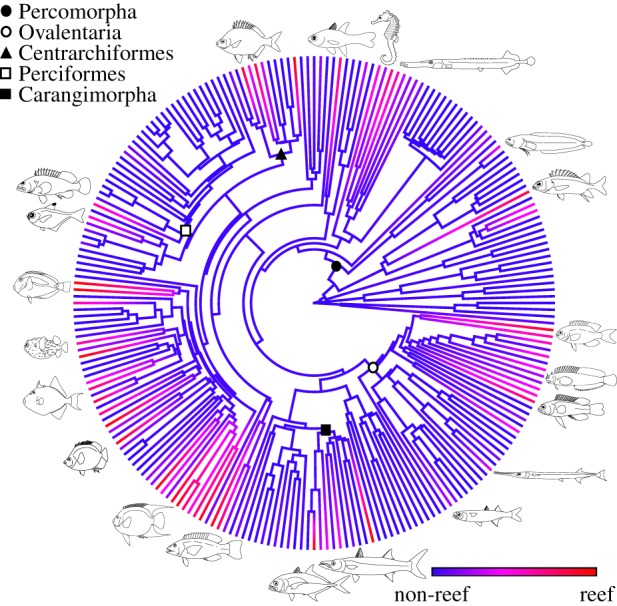
Inferred history of reef-dwelling across acanthomorph families. For the purposes of illustration, the posterior probability of each lineage living in ‘reef’ (red = 100% reef) or ‘non-reef’ (blue = 100% non-reef) habitats was calculated [26] from 10 000 stochastic character maps [27] on the maximum clade credibility tree of acanthomorphs [24] pruned to a single representative of each family. Nodes of important clades are identified and the general morphology of the main reef fish families depicted by line drawings.

The historical influence of changing reef composition and the K–Pg extinction upon the assembly of the reef fish fauna has been a topic of considerable speculation but remains uncertain. Reef systems are known drivers of diversification and net exporters of biodiversity to other marine habitats [18]; several lineages of reef fishes have been shown to have higher rates of speciation than non-reef-dwelling lineages [19,20]. Spiny-rayed fishes (Acanthomorpha) make up the vast majority (approx. 92%) of modern reef fishes and today reefs support approximately one-quarter of acanthomorph diversity [21], representing nearly 4500 species in 133 families. Available evidence from both fossils and living species does not clearly indicate whether reefs acted as cradles of early acanthomorph diversity or were later colonized by lineages that then diversified. The deepest diverging acanthomorph lineages (e.g. Polymixiiformes, Percopsiformes, Zeiformes, Gadiformes and Lampriformes) and immediate outgroups of acanthomorphs (e.g. Myctophiformes) are not closely associated with reefs. However, the most ancient fossil acanthomorphs, from the early Late Cretaceous (*ca* 100 Ma), are known from a variety of depositional settings, some of which are interpreted as being located proximal to well-developed benthic communities, although perhaps not true reefs [22]. The earliest uncontested reef fish assemblage from Bolca, Italy (*ca* 50 Ma) contains eight of the 10 most common extant coral reef families [23], all of which are acanthomorphs. Until very recently the relationship between reef fish families was unclear owing to the lack of a well-resolved phylogeny of acanthomorphs but new trees [24,25] have confirmed that reef fishes are not monophyletic (figure 1). Thus, if reef lineages originated in other habitats, major colonization of reef habitats must have occurred between the origination of acanthomorphs, estimated at 133–152 Myr ago based on molecular clock analyses [24], and when they rose to ecological dominance 10 Myr after the K–Pg [28]. Indeed, both the Late Cretaceous [1,29] and early Palaeogene [23] have been suggested as periods of intensive reef invasion by acanthomorphs, with subsequent radiation of many acanthomorph lineages linked to the rise of scleractinian reefs in the early Palaeogene [1,29]. Most extant acanthomorph families originated in the Palaeogene [24] and it is conjectured that a coevolutionary arms race between corals and fishes lead to the major re-organization of reefs during the Eocene [30].

Apart from these broad and often speculative outlines, key details of the evolutionary assembly of the unique reef fish fauna remain unknown. In the absence of a dense fossil record of reef fishes, we used a recent time-calibrated phylogeny of all major lineages of living acanthomorphs [24] in order to infer the history of reef-living and disparity through time (DTT). We determine that reef-living evolved independently in multiple lineages and identify two waves of reef invasion on either side of the K–Pg boundary, which were accompanied by increasing morphological convergence. This pattern is consistent with a macroevolutionary niche-filling scenario [31]: early transitions to reefs were made rapidly by morphologically distinct lineages and were followed by a slowing in the rate of invasions and eventual saturation of morphospace.

## 2. Material and methods

### (a) Data collection

We generated a morphological dataset of 12 traits that summarize body shape and size and which reflect several prominent functional properties of the feeding and locomotor systems. Measurements were made on adults of one species from 191 of the 228 acanthomorphs families in the phylogeny, each exemplar species was carefully selected to represent the most common head–body shape and size within the family. Ten linear traits were measured in millimetres with hand-held dial callipers from three adult specimens of each species, including standard length, lower jaw length, length of the dentigerous arm of the premaxilla, length of the ascending process of the premaxilla, diameter of the eye, maximum body depth, maximum body width, depth of the caudal peduncle, span of the caudal fin, length of the dorsal fin, along with body mass weighed to the nearest 0.01 g on a scale. Finally, suction index, a metric of the relative capacity to generate suction pressure during suction feeding [32,33] was calculated from five additional measurements, including diameter of the mouth aperture, length of the buccal cavity, distance from the joint between the supracleithrum and post temporal bones to the midpoint of the buccal cavity, height of the epaxial musculature from the supracleithrum–post temporal joint, and width of the epaxial musculature between the supracleithrum–post temporal joints. Specimens were either part of P.C.W.'s personal research collection or part of the ichthyological collection at the California Academy of Sciences (morphological data provided in the electronic supplementary material, dataset S1). Finally, we produced a multivariate estimate of morphology using a phylogenetic principal components analysis (PCA) of all 12 traits (PCA loadings available in the electronic supplementary material, Results and methods table S1). Prior to the PCA, we log-transformed all linear measurements and masses were logged after cube-root transformation.

We used a binary habitat variable: ‘Reef’ (1) or ‘Non-Reef’ (0). To avoid an arbitrary threshold for assigning a habitat to each family, we used the percentage of extant species in each family that are reef-associated as given by FishBase [21] (this includes fishes living on or near any shallow-water, consolidated wave-resistant structure) using functions in the R package rfishbase [34]. These percentages were used to specify a binomial distribution from which we sampled 100 times for each family, generating 100 habitat datasets. Using this method, between 35 and 53 families were recognized as ‘reef’ (reef datasets provided in the electronic supplementary material, dataset S2).

To incorporate phylogenetic uncertainty, all analyses were run across a random sample of trees from the posterior distribution of time-calibrated trees (BPDT) generated by Near *et al*. [24]. Briefly, the trees were built using 10 protein-coding genes comprising 8577 base pairs. Each codon position was analysed separately using a GTR + G model. Divergence times were estimated using 37 fossil calibrations (only 10 were from Bolca; calibrations 13, 15–18, 22, 24, 30, 32–33 in [22]) with an uncorrelated lognormal model of molecular evolutionary rate heterogeneity implemented in the computer program BEAST v. 1.6.1 [35,36]. Analyses were run four times, each run consisted of 1.0 × 10^9^ generations and sampled every 10 000 generations. The resulting trees from each of the four runs were combined and the burn-in period was identified and discarded prior to sampling the 1000 trees.

Within these trees, taxonomic sampling is sparse at the species-level as they contain approximately 3% of extant acanthomorph species. However, family-level sampling is far more complete, including 228 out of a total of 322 extant acanthomorph families [37]. We therefore ran our analyses on family-level trees, with each species assigned to a family using Catalog of Fishes [37]. As some families were not always monophyletic, we randomly sampled a single exemplar species for each family on every tree used, then converted the tips of the trees to family-level taxa. The root ages, branch lengths and phylogenetic relationships in these trees will vary. Tree manipulations were completed in the statistical computing framework ‘R’ [38] using the packages ape [39] and geiger [40].

### (b) Timing of reef colonization

**Figure 2.**
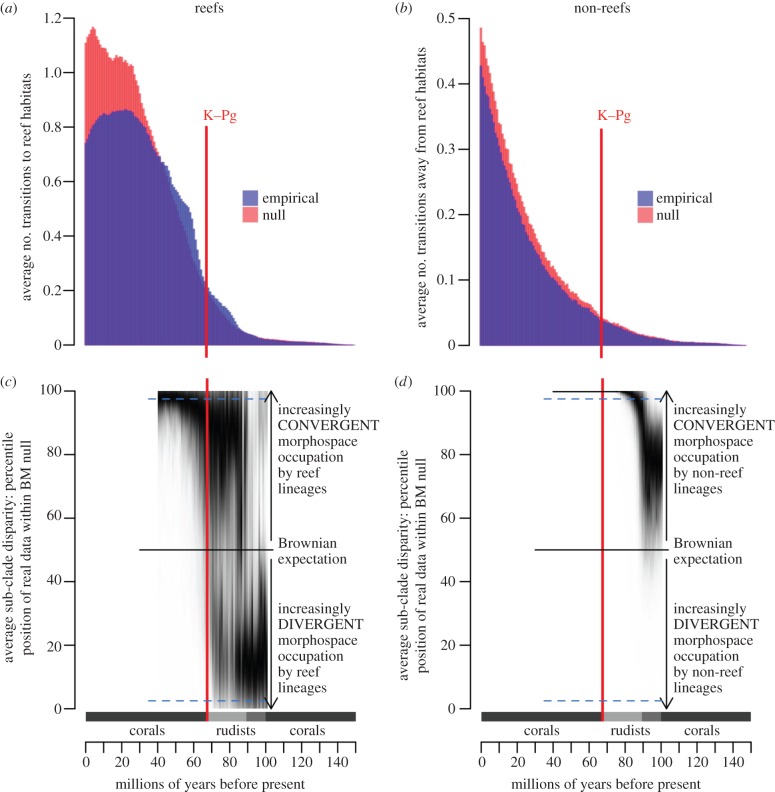
Patterns of movement on and off reefs and the evolution of morphological DTT. The primary bioconstructor during different periods is indicated on the *x*-axis with the changeover between corals and rudists occurring between 100 and 90 Ma [11]. The vertical red line indicates the timing of the K–Pg mass extinction. (*a*) Average number of transitions to reef habitats within acanthomorph fishes per million years, in the empirical data (blue) and the non-phylogenetic null (red). Results are averages across 50 000 stochastic character maps generated on a random sample of tree topologies from the Bayesian posterior distribution of trees. (*b*) Average number of transitions away from reef habitats within acanthomorph fishes per million years, in the empirical data (blue) and the null (red). (*c*) Density strip depicting the average sub-clade disparity of reef lineages, represented as the percentile position within the Brownian motion (BM) null per million years estimated on 1000 different reef acanthomorph phylogenies. The dashed blue horizontal lines represent the 95% confidence interval (CI). (*d*) Density strip depicting the average sub-clade disparity of non-reef lineages, represented as the percentile position within the Brownian motion (BM) null per million years estimated on 1000 different reef acanthomorph phylogenies. The dashed blue horizontal lines represent the 95% CI. Resolution in (*b*,*c*) is restricted to 100–40 Myr ago, which is when the majority of branching events occur within the trees. Density strips [42] can be thought of as a two-dimensional representation of three-dimensional histograms for each time bin, where a darker colour indicates a higher density of points and thus a higher bar in the histogram.

To estimate the number of transitions to reef-living through time, we used stochastic character mapping [41] in SIMMAP [27] with a uniform prior on the symmetry of the transition rate matrix (*α* = 1 and *κ* = 101) and a branch length prior on the rate parameter, to generate a total of 50 000 stochastically mapped trees. For each of the 100 habitat datasets, we sampled 50 trees from the BPDT and generated 10 stochastic character maps on each tree (provided in the electronic supplementary material, dataset S3). We wrote an R script [38] to convert these mapped trees into a distribution of absolute ages for the on (0–1) and off (1–0) reef transitions (R function available in the electronic supplementary material, dataset S4). To ensure that the patterns were not generated by the phylogenetic structure of the trees, we generated a null distribution by re-shuffling the habitat data on the tips of the phylogeny and re-running the analyses. To produce the same number of null SIMMAP trees as we had in our empirical dataset (50 000), we randomly resampled without replacement each of the 100 habitat datasets five times, generating 500 null habitat datasets. Then, for each null dataset, we sampled 10 trees from the Bayesian posterior distribution and generated 10 stochastic character maps per tree. The null stochastic maps were then analysed in the same way as the real data. Reef transitions are shown in figure 2*a* and non-reef in figure 2*b*. For further details, see the electronic supplementary material, Results and methods.

### (c) Phylogenetic clustering of reef habitat

Phylogenetic clustering can be inferred from our SIMMAP analyses which compare the empirical number of transitions in our data to a null generated by shuffling the data on the tips of the tree (sometimes referred to as a non-phylogenetic null). This is the test for phylogenetic inertia in discrete data suggested by Maddison & Slatkin [43] using stochastic mapping instead of ancestral state reconstruction via parsimony. Clustering is indicated by fewer transitions in the empirical data compared with the non-phylogenetic null. To confirm the phylogenetic clustering results from the stochastic character mapping, we also calculated the net relatedness index (NRI) [44] across the 1000 phylogenies from the BPDT and 100 habitat datasets using the R package picante [45]. NRI reflects the phylogenetic structure across the phylogeny and is calculated as the mean pairwise distance, as measured by the branch length, among all pairs of reef species and compared to a null where the data are reshuffled on the tips of the phylogeny.

### (d) Disparity through time

We sampled 10 trees from the BPDT for each of the 100 habitat datasets and pruned the trees into reef and non-reef species. With the morphological PCA axes, we estimated DTT on the 1000 reef and non-reef trees separately using the methods of Harmon *et al*. [46] implemented in the R package geiger [40]. This method estimates the average squared Euclidean distance between species across the entire phylogeny and for every node in that phylogeny (sub-clade). Relative disparities for each sub-clade are calculated by dividing the sub-clade disparity by that for the whole phylogeny. Finally, at each node the mean relative disparity is calculated as the average of the relative disparities of all sub-clades whose ancestral lineages are present at that time. The null, unconstrained Brownian motion was generated by 1000 simulations with the same variances as the 12 PC axes for each phylogeny. To combine the results across different tree topologies, we converted the average sub-clade disparity from the DTT analysis from relative to absolute time. Then, for each 1 Myr time bin, we calculated the percentile position of the real data within the Brownian motion null distribution. Therefore, values close to 100 indicate evidence of increasing disparity within clades and overlapping morphospace occupation, and close to 0 indicate increasing disparity between clades, which occupy different areas of morphospace. The percentile position of the real data across the 1000 trees are visualized using density strips [42] implemented in the R package denstrip, with the density constrained to vary between 0 and 100. You can think of these density strips as a two-dimensional representation of three-dimensional histograms for each time bin, where a darker colour indicates a higher density of points and thus a higher bar in the histogram. If disparity was evolving according to the null Brownian motion model, this graph would have a dark bar around the 50th percentile with the colours fading to grey away from it—representing a normal distribution. For further details, see the electronic supplementary material, Results and methods.

## 3. Results

### (a) Timing of reef colonization

The stochastic character maps show that reefs were successively invaded by multiple acanthomorph lineages and indicate two clear periods of elevated reef transitions. The first in the Late Cretaceous from 90 to 72 Ma and the second immediately following the K–Pg extinction (65–56 Myr ago) (figure 2 and electronic supplementary material, Results and methods figure S1). Neither period of increased reef invasion can be explained by rapid lineage diversification because the empirical number of transitions substantially exceeds the null that incorporates the impact of tree shape (figure 2*a*). Additionally, there is no evidence of elevated rates of taxonomic diversification in acanthomorphs following the K–Pg [24]. Beginning in the late Eocene (39 Ma), the average number of reef transitions drops below the non-phylogenetic null expectation, indicating that reef lineages become phylogenetically clustered from this period onward [43]. For the reef to non-reef transitions, the pattern in the empirical data mirrors the non-phylogenetic null, suggesting that the number of transitions off reefs is mainly determined by the number of lineages in each period (figure 2*b*).

### (b) Phylogenetic clustering of reef habitat

**Figure 3.**
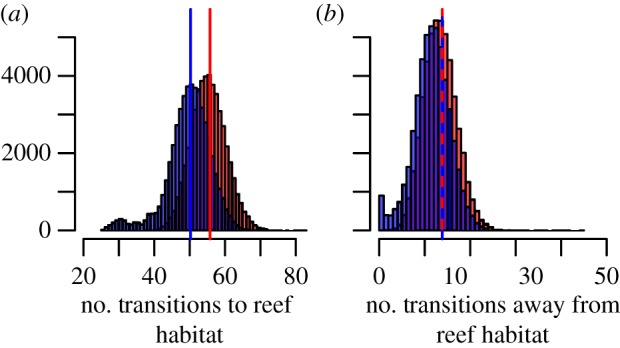
Estimated number of transitions to and from reef habitats within acanthomorph fishes. (*a*) Histogram of the estimated number of transitions from non-reef to reef habitat in the real data (blue) and the non-phylogenetic null (red). (*b*) Histogram of the estimated number of transitions from reef to non-reef habitats in the real data (blue) and the non-phylogenetic null (red). Estimated using a total of 50 000 stochastic character maps generated in SIMMAP [27]. The vertical lines indicate the average number of transitions. These distributions illustrate the variability in the number of transitions owing to the uncertainty in the character mapping as well as the tree topology, branch lengths and reef-living.

In total, we infer about 50 independent transitions to reef-dwelling generated the acanthomorph reef fish fauna we see today (figure 3), confirming that far from being a monophyletic group from a single invasion, modern reef fishes represent a diverse assemblage accumulated through many invasions. Overall, we estimate fewer reef transitions from the empirical data than the non-phylogenetic null model (figure 3*a*) [43], indicating reef lineages are, on average, phylogenetically clustered. This result is supported by the NRI, with negative values of the standardized effect size and the low *p*-values confirming that reef lineages are more closely related than expected by chance (see the electronic supplementary material, Results and methods figure S2). By contrast, transitions away from reefs follow the null expectation suggesting that the lineages which move away from reefs are not phylogenetically clustered (figure 3*b*).

### (c) Disparity through time

High average sub-clade disparity relative to the null indicates that reef lineages independently converge on similar morphologies, while low average sub-clade disparity indicates that lineages occupy distinct regions of morphospace. We find that reef (figure 2*b*) and non-reef (figure 2*c*) lineages show different patterns of morphospace occupation through time. Early in the evolutionary history of acanthomorphs, non-reef lineages exhibit substantial phenotypic overlap, but lineages that invaded reef habitats early occupy distinct regions of morphospace. Beginning in the Late Cretaceous (*ca* 85 Ma), there is a shift to greater morphological convergence within sub-clades in both reef and non-reef lineages. By 80 Ma, the Brownian motion model is rejected for non-reef lineages as all results exceed the 97.5th percentile, indicating that all independent non-reef lineages occupy similar regions of morphospace. By contrast, reef lineages do not start to exhibit such strong morphological convergence until the early Eocene (50 Ma), well after the K–Pg mass extinction.

## 4. Discussion

Our results reveal that reef lineages of acanthomorphs successively colonized reef habitats throughout the Late Cretaceous and early Palaeogene. Thus, in contrast to marine benthic invertebrate genera [18], reefs appear not to have been cradles of higher level fish diversity (family and above). This is consistent with the little we can infer from the fossil record, as the oldest acanthomorph fossils are found in a range of depositional settings. There is also no evidence that familial diversity was systematically exported to other habitats, as the transition rates away from reefs fit the null expectation (figure 3*b*). However, we stress that these results are at the family-level, most reef families contain both reef and non-reef species, so transitions on and off reefs will have been far more dynamic over the history of acanthomorphs than we can infer. Reefs have clearly been important drivers of both lineage and morphological diversification within fishes [19,20,47] and may be acting as cradles and sources of diversity within families.

There is remarkable congruence between the temporal pattern of changes in the number of transitions onto reefs, morphospace occupation by reef lineages and reef history. Around 90 Ma, the number of transitions to reef habitats first exceeds the null expectation and a little later (approx. 85 Ma) morphospace occupation in reef lineages shifts from sub-clades occupying primarily distinct areas to overlapping regions. Later, following the K–Pg mass extinction, we identify a second rapid rise in the number of reef transitions accompanied by a move towards even greater overlap in morphospace occupation by reef sub-clades. By approximately 50 Ma, reef lineages occupied entirely convergent areas of morphospace (figure 2*c*) and the number of reef transitions begins to plateau. For the last 30 Myr, we estimate far fewer transitions to reef habitats than expected under the null model (figure 2*a*). This sequence of events indicates that early transitions to reefs were by phylogenetically over-dispersed lineages but from 30 Ma onwards, reef lineages are phylogenetically clustered. As most modern acanthomorph families had evolved by this time [24,25] this result suggests that reef families cluster upon the phylogeny, a conclusion supported by the overall number of transitions (figure 3*a*) and the NRI results.

Our pattern of early reef colonization by morphologically distinct lineages, later pulses of invasion accompanied by increasingly convergent morphospace occupation culminating with morphospace saturation and a plateau in reef invasions, is consistent with a macroevolutionary niche-filling scenario [31]. Niche-filling, or diversity-dependent models (e.g. [48]), are typically associated with monophyletic radiations. When ecological opportunity is high, there is a rapid increase in disparity and species diversity, which slows over time as niches become saturated. The increase in species diversity during adaptive radiation is often attributed to elevated rates of speciation, but in our case, reef fish diversity increases through more rapid colonization of reef habitats.

The two waves of niche filling on either side of the K–Pg boundary coincide with, and might be linked to, changes in reef structure and climate. The Late Cretaceous is a ‘greenhouse’ interval lacking permanent icecaps at the poles [10]. Elevated sea surface temperatures during the Cretaceous have previously been correlated with increased richness of marine fishes [49]. Fluctuating sea-levels and reef volume in the low to mid-latitudes of the Northern Hemisphere during the Late Cretaceous are also associated with a volatile shift from corals to rudist bivalves as the principal bioconstructors of reefs [10,11]. A collapse in rudist-bearing carbonate platforms at the Cenomanian–Turonian boundary (*ca* 93 Ma) [50] was followed by the re-establishment of rudist diversity over the next 10 Myr [11]. It is possible that enhanced ecological opportunity through vacant niches have given acanthomorphs opportunities to invade reefs in ways not possible in the face of more established, mature communities. Indeed, Late Cretaceous fossil acanthomorphs are found on carbonate platforms associated with rudist reefs and include early tetraodontiforms and syngnathiforms, groups which today are associated with coral reefs ([29] and references therein).

The second wave of colonization following the K–Pg mass extinction is associated with the recovery and expansion of scleractinian coral reefs in the early Palaeocene [15,30]. Coral diversity decreased during the K–Pg mass extinction but there was no substantial decline in reef carbonate production [13]. Corals recovered in the earliest Palaeocene (Danian, 65.5–61.7 Myr ago): coral formations were more widespread than in the Late Cretaceous [15,16] and over one-fifth of Danian coral genera were new [13]. Rudist reefs of the Late Cretaceous were simple [11] and it has been argued they should not be considered true reef-builders [12], whereas the mound and framework reefs of the early Palaeogene were clearly more complex structures. Therefore, Palaeocene acanthomorphs, after gaining a foothold in unstable reefs and reef-like structures of the Late Cretaceous, were confronted by more structurally complex reef ecosystems that might have fuelled a second wave of invasion and innovation. Within 10 Ma of the K–Pg, reef invasions had slowed, this could relate to the decline of reef volume associated with the PETM [13,49], the saturation of available niches or some combination of the two. Overlapping morphospace occupation between reef fish sub-clades in the early Eocene (*ca* 50 Ma) suggests the saturation of ecomorphological niches on reefs and the origination of most functional groups within reef-dwelling acanthomorphs. The onset of ecomorphological exhaustion corresponds in time to the exceptional palaeontological window provided by the Bolca *Lagerstätte*, which confirms the existence of an ecologically diverse reef fish fauna including the earliest herbivorous acanthomorphs [23,51].

Reef fishes are one of the most diverse vertebrate assemblages on Earth, and their complex evolutionary history is rapidly being illuminated by improved phylogenetic resolution and continued palaeontological efforts. The correspondence between our phylogenetically inferred results and the existing fossil record suggests that the dynamic history of reefs had a profound impact on the origin and early assembly of the modern reef fish fauna. The colonization of reefs by the ancestors of modern reef fishes may have been promoted by ecological opportunity associated with unstable Late Cretaceous reefs that was amplified by the rapid rise of structurally complex scleractinian coral reefs after the K–Pg mass extinction. The influence of reefs may continue into the present as previous work has found high rates of speciation [20] and morphological diversification [47] in several reef-dwelling fish lineages when compared with non-reef species. This remarkable synergy between reefs and acanthomorph fishes, which has evolved over the last 100 Myr, has resulted in a complex, interconnected evolutionary history that is currently being eroded by overfishing and degradation of coral habitats by pollution and rising sea temperatures.

## Notes

This study meets the terms of the ethics committee at the institution where the experiment was carried out.

## Data accessibility

All datasets are presented as the electronic supplementary material.
